# Notch Signaling in T-Cell Development and T-ALL

**DOI:** 10.5402/2011/921706

**Published:** 2011-01-23

**Authors:** Xiaoyu Li, Harald von Boehmer

**Affiliations:** Department of Cancer Immunology and AIDS, Dana-Farber Cancer Institute, Harvard Medical School, 44 Binney Street, Boston, MA 02115, USA

## Abstract

The Notch signaling pathway is an evolutionarily conserved cell signaling system present in most multicellular organisms, as it controls cell fate specification by regulating cell proliferation, differentiation, apoptosis, and survival. Regulation of the Notch signaling pathway can be achieved at multiple levels. Notch proteins are involved in lineage fate decisions in a variety of tissues in various species. Notch is essential for T lineage cell differentiation including T versus B and *αβ* versus *γδ* lineage specification. In this paper, we discuss Notch signaling in normal T-cell maturation and differentiation as well as in T-cell acute lymphoblastic lymphoma/leukemia.

## 1. Notch Signaling Pathway

The Notch gene was initially described in a mutant strain of fruit fly Drosophila melanogaster with notched wing blades in 1917 by Morgan [1]. This notched wing phenotype resulted from a partial loss of function of the Drosophila Notch gene, which was cloned and characterized in the mid-1980s [2, 3]. The Notch signaling pathway is an evolutionarily conserved cell signaling system present in most multicellular organisms, as it controls cell fate specification by regulating cell proliferation, differentiation, apoptosis, and survival [4]. Mammals possess four transmembrane Notch receptors (Notch1, 2, 3, and 4), [5–10] which can interact with five Notch ligands (Delta-like 1 (DL1), Delta-like 3 (DL3), Delta-like 4 (DL4), Jagged1 and Jagged2) [11–16]. The Notch receptors are synthesized as single protein which are proteolytically cleaved in the Golgi by a furin-like protease at site S1 during transport to the cell surface, resulting in heterodimers consisting of the extracellular domain and the transmembrane domain followed by the intracellular domain of the Notch receptor [17]. The extracellular domain of Notch contains 29–36 epidermal growth factor- (EGF-) like repeats (36 in Notch1 and Notch2, 34 in Notch3, and 29 in Notch4) that are responsible for ligand binding interactions [18]. The EGF-like repeats are followed by three cysteine-rich LIN12-Notch repeats (LNRs) that prevent ligand-independent activation of Notch signaling pathway [19, 20] and the heterodimerization domain (HD). The intracellular domain of Notch contains an RAM23 domain followed by six ankyrin repeats (ANK) involved in binding to the CSL transcription factor [21, 22], a transactivation domain (TAD), and a PEST motif (polypeptide enriched in proline (P), glutamic acid (E), serine (S), and threonine (T)) that regulates protein stability [23, 24].

Ligand-receptor interaction between neighboring cells triggers two successive proteolytic cleavages of the receptor, which release the intracellular portion of the Notch receptor (ICN) from the plasma membrane. The first cleavage is mediated by ADAM (a disintegrin and metalloprotease) proteases at site S2, located on the extracellular side, about 12 amino acids away from the transmembranse domain, which is a key regulatory step in Notch activation [20]. The second proteolytic cleavage is mediated by the *γ*-secretase complex containing presenilin and nicastrin within the transmembrane domain at site S3. This releases intracellular domain of Notch which then translocates to the nucleus, where it binds to the DNA-binding transcriptional factor CSL [25, 26] (known as CBF1 in human, suppressor of hairless in Drosophila, LAG in Caenorhabditis elegans, and also called RBP-J*κ* in mice). In the absence of ICN, CSL represses transcription by binding to the promoters of target genes and recruiting histone deacetylases and corepressors such as SMRT/NcoR and SHARP/MINT/SPEN [27, 28]. When ICN binds to CSL, it recruits the coactivators including Mastermind-like1 (MAML1) [29, 30] and histone acetyltransferase [31], which binds to ICN in the ICN-CSL-DNA complex, thereby converting the CSL complex into a transcriptional activator. 

## 2. Modulation of Notch Signaling

Regulation of Notch signaling pathway can be achieved at multiple levels. First, Fringe proteins have been identified as Golgi-localized glycosyltransferases that inhibit Notch signaling by interfering with Notch receptor-ligand interactions through glycosylation [32–36]. Koch and co-workers showed that T cell specific ectopic expression of Lunatic Fringe decreases T-cells and induces lymphoid progenitor to adopt the B-cell fate in the thymus [37]. Second, the *γ*-secretase inhibitors (GSIs) effectively block activation of Notch receptor by preventing proteolytic cleavage at site S3, releasing the Notch intracellular domain (ICN) [38]. It has been shown that treatment of T-ALL cells with the GSI results in cell cycle arrest followed by apoptosis [39]. Third, MAMLs are important for Notch-dependent CSL transcriptional activation [29, 30]. Dominant negative mutants of MAML are capable of inhibiting Notch signaling since DNMAML1 contains only the N-terminal ICN-binding basic domain which allows them to bind to ICN but lacks the activation domain therefore, DNMAML1 antagonizes Notch1 signaling by inhibiting recruitment of transcription coactivators [40–42]. Studies from the Pear group show that expression of DNMAML1 leads to marked inhibition of early T-cell differentiation and to the appearance of intrathymic B cells, phenotypes consistent with inhibition of Notch1 [43]. Fourth, Fbw7 is the F-box component of an SCF-E3 ubiquitin ligase complex (SCF^FBW7^). Fbw7 targets Notch1 for ubiquitination and degradation [44–46]. The PEST domain in the C terminus of the Notch1 receptor is essential for phosphorylation-mediated and proteasome-dependent degradation of Notch1, because Fryer et al. reported that CycC:CDK8 phosphorylates the ICN1 within the TAD and PEST domains, and CycC:CDK8 expression strongly enhances ICN1 hyperphosphorylation and PEST-dependent degradation by the Fbw7 *in vivo* [47], suggesting that Notch1 protein stability could be a critical regulator of intracellular signaling thresholds. 

## 3. Notch in T-Cell Development

### 3.1. T versus B Lineage

Notch proteins are involved in lineage fate decisions in a variety of tissues in various species [4]. Notch is essential for T lineage cell differentiation. Loss-of-function experiments have shown that Notch plays a crucial role in determining T lymphoid versus B lymphoid lineage decision [48–50]. Conditional deletion of Notch1 in hematopoietic progenitors induces a block of early T-cell development and accumulation of ectopic immature B-cells in the thymus [49, 50]. Similar phenotype was observed in the mice harboring Cre-mediated deletion of RBP-J created by Han et al. [48]. Moreover, interference with Notch receptor-ligand interactions or inhibition of Notch-mediated transcription results in failure of T cell development and promotion of B cell development [37, 43]. Conversely, gain-of-function analyses involving overexpression of constitutively active Notch1 in bone marrow lineage negative progenitors indicate that activated Notch1 results in thymic-independent T-cell development at the expense of B-cell development in the BM and does not influence granulocyte maturation [51]. Nevertheless, other studies suggest that constitutive Notch1 activity blocks or delays myeloid differentiation concomitant with ectopic T-cell development [52–55]. Thus, whether or not activated Notch1 affects myeloid differentiation remains controversial. Taken together, these findings demonstrate that Notch1 singaling is essential to induce T-cell lineage commitment from the multipotent hematopoietic progenitor cells (HPCs). 

### 3.2. Early T-Cell Development

The earliest intrathymic T-cell precursors, ETPs, which are characterized by high expression of c-Kit receptor and low expression of the interleukin 7 receptor alpha chain (IL7R), are found in the double-negative DN1 thymocyte subset (CD4^−^CD8^−^CD25^−^CD44^−^) [56–59]. Notch signaling is required not only for generation of the ETP population but also for transitions of ETP-to-DN2 and ETP-to-DN3 suggesting that Notch1 activation is needed continuously to promote survival or proliferation throughout the early stages of intrathymic T-cell development [58, 59]. Consistent with these findings, a number of *in vitro *studies provide supportive evidences showing that Notch receptor-ligand interactions are necessary for induction and maintenance of T-cell lineage specification at both the DN1 and DN2 stages of T-cell development [60, 61] and that enforced expression of Notch inhibitor Nrarp (Notch-regulated ankyrin-repeat protein) in mouse HSCs results in a profound block during progression through early stages of thymocyte maturation (DN1–DN3) [62]. 

### 3.3. *β*-Selection Checkpoint

There are two checkpoints during T-cell development, where cells are rescued from programmed cell death and progress in their development [63, 64]. Commitment to either the CD4^+^CD8^+^ *αβ* or CD4^−^CD8^−^ *γδ* lineage occurs at the first checkpoint [65]. DN3 cells that generate the productive TCR-*β* chain rearrangement which can be assembled into the pre-T-cell receptor (pre-TCR) complex consisting of a TCR*β* chain, the invariant pT*α* chain [66], and CD3 molecules can survive the transition from DN3 to DN4, this process is termed *β* selection [67–69]. Loss of RAG-1 or RAG-2 *in vivo* results in total inability to initiate V(D)J rearrangement, leading to the arrest of developing *αβ* lineage thymocytes at the DN3 stage [70, 71]. At the second checkpoint in T-cell development, cells on the *αβ* lineage are rescued from cell death by binding of their *αβ* TCR to thymic MHC molecules, allowing CD4^+^CD8^+^ thymocytes to undergo positive and negative selection [63, 64]. Wolfer and colleagues reported that inactivation of Notch1 prior to the DN3 stage severely impairs *αβ* but not *γδ* T-cell development and partially blocks thymocyte development at the pre-TCR checkpoint due to a severe impairment of V*β*-DJ*β* rearrangement [72]. Conditional ablation of the gene encoding RBP-J at an earlier developmental stage results in enhanced generation and accelerated emigration of *γδ* T cells, whereas *αβ* T-cell development is arrested at the DN3 stage [73]. Inhibition of Notch signaling by DNMAML in DN3 stage leads to similar consequences [40]. Moreover, the OP9-DL1 in vitro T-cell differentiation system provides an ideal system to mimic *in vivo* T-cell development established by Zúñiga-Pflücker and colleagues [74, 75]. They made use of OP9-DL1 system to demonstrate that pre-TCR signaling concurrent with Notch receptor-ligand interactions are required for productive *β* selection outcomes including rescue from apoptosis, proliferation, and transition of DN thymocytes to the DP stage of T-cell development [76–78]. 

### 3.4. *αβ* versus *γδ* Lineage

In vitro experiments provided evidence that bifurcation of the *αβ* and *γδ* T-cell lineages is completed at the DN3 stage [77, 79–82]. Robey and colleagues examined the effect of Notch activity and the T-cell receptor in the *αβ* versus *γδ* T-cell lineage choice. They showed that cells with Notch^+/−^ gene are less likely to become *αβ* T-cells than wild type cells with Notch^+/+^ gene that develop in the same mouse. In addition, they also provided evidence that *γδ* to *αβ* T cells are consistently higher in T cells derived from Notch^+/−^ stem cells compared to wild-type cells, implying that reduced Notch activity favors the *γδ* lineage over the *αβ* lineage. We have shown that *γδ*TCR-expressing cells can survive and expand in the absence Notch signals [83]. Mice deficient in Jagged2 exhibit altered thymic morphology and impaired differentiation of *γδ* lineage T cells [84], suggesting that Jagged2-mediated Notch signaling participates in *γδ* lineage differentiation. However, the role of Notch signals during *αβ* versus *γδ* T lineage decision remains controversial. Experiments by Wolfer and colleagues demonstrate that conditional deletion of Notch1 signaling at DN2-DN3 developmental stage leads to severe perturbation of *αβ* but not *γδ* lineage development [72]. In fact, Garbe et al. [83]as well as Ciofani et al. [77] demonstrated a stage-specific requirement for Notch signaling at the *αβ* and *γδ* T lineage bifurcation. 

## 4. Notch Signaling in T-ALL

The involvement of Notch1 was first observed in the t(7;9)(q34;34.3) translocation, a rare recurrent chromosomal rearrangement present in <1% of human T-ALL patients. The t(7;9)(q34;34.3) translocation juxtaposes the C-terminal region of EGF repeat 34 of the human NOTCH1 gene next to the TCR*β* promoter/enhancer, resulting in the activated expression of NOTCH1, which was named TAN1 for translocation-associated Notch homolog [5]. TAN1 was shown to function as a specific oncoprotein for T cells in the murine bone marrow transplantation model. The murine tumors induced by TAN1 in this model are phenotypically similar to the TAN1-associated human tumors [85]. Notch was originally thought to play a minor role in the molecular pathogenesis of human T-ALL due to the rare frequency of t(7;9)(q34;34.3) translocation involving Notch in human T-ALL cases. More recently, it was shown that more than 50% of human T-ALLs, including tumors from all major molecular oncogenic subtypes, have gain-of-function mutations that involve the extracellular heterodimerization domain and/or the C-terminal PEST domain of NOTCH1 [86]. Following this finding, activating mutations of Notch1 as well as mutants of genes that regulate turnover of intracellular Notch1 have been detected in mouse models of T-ALL [44, 45, 87–89]. Thus, these studies have sparked renewed interest in the pathogenesis of T-ALL and greatly expanded the role of NOTCH1 in the etiology and molecular tumorigenesis of this human disease with the hope of finding novel targeted therapies that interfere with NOTCH signaling. 

### 4.1. E2A Inhibition

E-proteins are members of highly conserved basic helix-loop-helix (bHLH) family of transcription factors which plays a critical role in cellular differentiation. The E2A gene encodes two alternatively spliced transcripts which produce two proteins, E47 and E12 [90–92]. These proteins bind as homo- or heterodimers with other basic helix-loop-helix proteins as transcription factors to E-box consensus sequences and have an essential function in B-cell and T-cell development [93, 94]. E2A-deficient mice lack B cells and regularly develop T-ALL [95]. Notch inhibition of E47 was initially described by Ordentlich et al. [96] who demonstrated that Notch and Notch downstream target Deltex act on E2A-encoded E47 by inhibiting signaling through Ras. Two mechanisms of E2A inhibition by Notch have been reported: Notch-mediated upregulation of the pre-TCR signaling leads to ERK-MAPK-dependent upregulation of the E2A inhibitors Id1 or Id3 which prevent binding of E2A-encoded proteins to E-box motifs [93, 97]. Alternatively, overexpression of Notch induces E2A protein degradation through ubiquitination-proteasome-mediated pathway both in vitro and *in vivo* [98, 99]. Most T-ALLs induced by other oncoproteins such as TAL1, LMO1, or LMO2 are characterized by inhibition of the transcriptional activity of the E2A proteins [100–105], suggesting that E2A could be an essential pathway in the leukemogenesis of T-ALL. In order to evaluate the role of E2A-encoded E47 protein as a potential tumor suppressor in murine T-ALL, we have introduced nondegradable mutant E47 by retroviral vector into intracellular domain of Notch1- (ICN1-) overexpressing tumors, and we found that overexpression of E47 led to suppression of tumor cell growth [98]. Taken together, these findings suggest that E2A may function as tumor suppressor, and inhibition of E2A activity is a common and critical event in the pathogenesis of T-cell lymphoma. 

### 4.2. c-Myc Activation

The c-Myc proto-oncogene is a basic helix-loop-helix leucine zipper (b/HLH/LZ) protein involved in cellular growth and differentiation. c-Myc has been identified as a critical direct downstream target gene of NOTCH1 in leukemogenesis [89, 106, 107]. Inhibitors of c-Myc prevent Notch1 from rescuing T-ALL cells treated with *γ*-secretase inhibitor (GSI), and overexpression of c-Myc is sufficient to rescue most human T-ALL cell lines from GSI-induced growth arrest [107]. Palomero and co-workers showed that Notch1 controls a feed-forward-loop transcriptional network that regulates cell growth and proliferation and that Notch1 directly activates multiple biosynthetic routes and targets c-Myc using integrating gene expression array and ChIP-on-ChIP analysis [106]. Sharma and colleagues developed doxycycline- (Dox-) regulated Notch1-IC mouse T-ALL cell lines and identified c-Myc as a direct Notch1 target gene in Notch1-induced T-ALL transformation using gene expression profiling and chromatin immunoprecipitation (ChIP) analysis in this cell line [89]. Moreover, Notch1 inhibition leads to cell cycle arrest and decreases c-Myc mRNA levels [89]. The results are consistent with those in human T-ALL studies [106, 107], implying that the direct activation of c-Myc mediated by Notch1 is required to maintain leukemic growth. To evaluate the mechanism by which Notch1 directly targets c-Myc, Satoh et al. [108] have demonstrated that activated forms of Notch1 and its downstream effector CSL can bind to the responsive element (TTCCCAA) located between −195 bp and −11 bp of the c-Myc promoter by luciferase reporter assay and electrophoretic mobility shift assay (EMSA). The recruitment of Notch1 to the CSL-binding sites in the c-Myc promoter has been confirmed by other investigators [89, 106, 107, 109] by luciferase reporter assay, EMSA, or chromatin immuno-precipitation (ChIP) analysis. We have previously systematically investigated oncogenesis of T-ALL initiated by ICN1 overexpression in a bone marrow transplantation (BMT) model; we found that c-Myc is upregulated in nonmalignant ICN1-overexpressing cells as well as malignant ICN1-overexpressing cells at both of mRNA and protein levels [98]. Furthermore, we have shown that deletion of c-Myc at the CD4^+^CD8^+^ stage of T cell development prevented tumor formation induced by Notch1 [98]. Girard and colleagues performed provirus insertional mutagenesis in c-Myc transgenic mice to identify c-Myc collaborators in leukemogenesis; they observed that Notch1 was mutated by provirus insertion upstream of the exon coding for the transmembrane domain of Notch1, resulting in high expression of truncated Notch1 RNAs and proteins [110]. Moreover, in collaboration with the Look lab, we have reported that dysregulated Myc expression is shared between ICN1-induced murine model and most human T-ALLs with Notch1 gene mutations [111]. These results suggest a collaboration of c-Myc and Notch1 in oncogenesis. 

### 4.3. Cell Cycle Progression

Cell cycle progression is regulated at several checkpoints of cellular process. Notch signaling has been shown to be a potent regulator of cell cycle progression in T-ALL cells [86, 89, 112]. Sicinski and colleagues [113] have studied the function of cyclin D3 in T-cell development and T-cell leukemogenesis, and they found that cyclin D3^−/−^) mice show impaired expansion of immature T lymphocytes, characterized by a marked deficit of CD4^+^CD8^+^ T cells. Cyclin D3 deficiency inhibits T-ALL induced by Notch1, suggesting the requirement of cyclin D3 for the growth of T-ALL tumors derived from immature T cells. To evaluate the mechanism by which Notch1 regulates cell-cycle progression in T-ALL, experiments from Joshi group [114] show that cyclin D3 functions together with its catalytic partners CDK4 and CDK6 to facilitate cell cycle progression in Notch1-dependent T-cell lymphoma, because Notch1 binds directly to the region of −1764 bp to −1537 bp of cyclin D3 promoter and specifically regulates cyclin D3 promoter activity revealed by ChIP analysis and luciferase reporter assay. Inhibition of Notch activation with GSI treatment abrogates the occupancy of Ntoch1 to the cyclin D3 promoter, suggesting that cyclin D3 is a direct target of the Notch/CSL signaling pathway [114]. While cyclin D3 expression contributes to cell-cycle progression in Notch-mediated human T-ALL cell lines, overexprssion of CDK4 or CDK6 together with cyclin D3 partially rescues these cell lines from GSI-induced G1 arrest. Moreover, cyclin D3 and CDK4 are highly expressed in Notch-dependent leukemic mice [114], which is consistent with our previous report [98]. Cyclin-dependent kinases (CDKs) and cyclin-dependent kinase inhibitors (CKIs) play important roles in the regulation of G1/S phase transition [115]. Sarmento [116] and colleagues provided the first evidence that Notch signaling links to the F-box protein SKP2, which is the subunit of the E3-ubiquitin ligase SCF^SKP2^that degrades p27^Kip1^ and p21^Cip1^. Their data showed that Notch signaling pathway specifically degrades p27^Kip1^ and p21^Cip1^ by directly inducing CSL-dependent transcription of SKP2, which subsequently enhances CDK2 kinase activity and accelerates cell cycle entry into S phase. Depletion of SKP2 by siRNA in G1 phase stabilizes p27^Kip1^ and p21^Cip1^, abolishes Notch effect on G1-S progression, and results in a significant reduction of cells in S phase [116]. This is consistent with observations from other groups demonstrating that GSI inhibition of Notch signaling in human T-ALL cell lines results in G0/G1 cell cycle arrest by downregulation of SKP2, upregulation of p27^Kip1^, and derepression of RB [117, 118]. 

### 4.4. ARF-Mdm2-p53 Pathway

The ARF-Mdm2-p53 tumor surveillance pathway is important for Myc-mediated apoptosis in primary mouse embryofibroblasts (MEFs) [119], whereas this pathway is frequently inactivated in Myc-induced lymphoma *in vivo* [120]. To evaluate whether or not c-myc-ARF-Mdm2-p53 axis is intact in ICN1-dependent tumor cells, we have shown that p53 and p19^arf^ were downregulated and Mdm2 was upregulated at both mRNA and protein levels in the tumor cells compared with nontumorigenic ICN1-overexpressing cells [98]. This study indicates that dysregulation of the c-Myc-p53 axis occurs in the face of genomic stability as revealed by spectral karyotype (SKY) and array-base comparative genomic hybridization (aCGH). Consistent with these observations, the Beverly group [121] reported that Notch suppresses p53 in lymphomagenesis in Notch1 transgenic mice, and p53 is activated upon inhibition of Notch signaling by GSI. They further proposed that Notch suppresses p53 in lymphomagenesis through repression of the ARF-Mdm2-p53 pathway. 

### 4.5. PTEN/PI3K/Akt-mTOR Pathway

To identify novel pro-oncogenic pathways regulated by Notch, Chan et al. [122] used reverse phase protein (RPP) microarrays to profile the phosphorylation changes in a large number of signaling proteins in 13 T-ALL cell lines treated with GSI they found that the phosphorylation of multiple signaling proteins in the mTOR pathway was suppressed by inhibition of Notch signaling in a Notch-dependent manner, as this phenomenon can be rescued by expression of ICN1 and mimicked by dominant negative MAML1, suggesting that Notch signals positively regulate activity of the mTOR pathway in T-ALL. More importantly, the effect of GSI on the mTOR pathway can also be rescued by c-Myc because c-Myc is a direct transcriptional target of Notch. Inhibition of mTOR by rapamycin combined with GSI treatment synergistically inhibited T-ALL cell growth [122]. Consistently, the work from other group showed that Notch1 signaling confers chemoresistance in a wild-type p53-dependent manner. Notch inhibited p53 through the PI3K-Akt/protein kinase B- (PKB-) mammalian target of rapamycin (mTOR) pathway, and the inhibition of this pathway reversed the chemoresistance [123]. PTEN, a tumor suppressor which negatively regulates PI3-kinase-Akt signaling pathway, is consistently downregulated in GSI-resistant T-ALL cell lines [124]. The Palomero group found that transcriptional downregulation of PTEN mediates physiologic upregulation of the PI3K-AKT pathway; mutational loss of PTEN and aberrant Akt activation induce resistance to GSI in T-cell leukemia [124]. In contrast to these findings, Medyouf et al. [125] reported that T-ALLs remain dependent on Notch signaling and found no correlation between PTEN status and resistance to Notch inhibition in primary human T-ALL and mouse model of T-ALL. 

## 5. Conclusions and Perspectives

Large numbers of work over the last decade have demonstrated that Notch signaling is essential for T-cell lineage fate decision, and aberrant activation of Notch signaling plays a critical role in etiology and pathogenesis of T-cell acute lymphoblastic Lymphoma/Leukemia. Activated Notch signaling is required for the transformation and growth of T-cell lymphoma/leukemia; downstream pathways that transmit pro-oncogenic signaling are poorly understood. In the mouse model, Notch1-mediated T-ALL develops monoclonal tumor; thus, another unknown secondary genetic event needs to be elucidated. Altered expression of miRNAs has been involved in various types of cancers including hematopoietic malignancies; however, the role of miRNAs in T-cell development and T-ALL tumorigenesis is not well characterized, although few related studies have been reported [126–128]. In the future, combination of GSI and downstream pathway regulators such as mTOR inhibitor or CyclinD3 inhibitor will represent a novel approach for treating this aggressive human malignancy. 
